# Toll-like receptors ligand immunomodulators for the treatment congenital diaphragmatic hernia

**DOI:** 10.1186/s13023-024-03384-7

**Published:** 2024-10-18

**Authors:** Mayte Vallejo-Cremades, Javier Merino, Rita Carmona, Laura Córdoba, Beatriz Salvador, Leopoldo Martínez, Juan Antonio Tovar, Miguel Ángel Llamas, Ramón Muñoz-Chápuli, Manuel Fresno

**Affiliations:** 1 IdiPAZ, Madrid, Spain; 2 Centro de Biología Molecular “Severo Ochoa”, CSIC-UAM, Madrid, Spain; 3 University of Málaga, Málaga, Spain; 4 Crazy Science SL, Mérida, Spain

**Keywords:** Fetal therapy, Toll-like receptors, Macrophages, Inflammation, Retinoic pathway, Embryonic development, Congenital diaphragmatic hernia, Orphan drug

## Abstract

**Background:**

Congenital diaphragmatic hernia (CDH) is a rare disease that affects the development of the diaphragm, leading to abnormal lung development. Unfortunately, there is no established therapy for CDH. Retinoic acid pathways are implicated in the ethology of CDH and macrophages are known to play a role in repairing organ damage.

**Methods:**

We have analyzed the effect of several Toll like receptor (TLR) ligands in the nitrofen-induced CDH model in pregnant rats widely used to study this disease and in the G2-GATA4^Cre^;Wt1^fl/fl^ CDH genetic mice model. Morphometric and histological studies were carried out. Immune cell infiltration was assayed by immunochemistry and immunofluorescence and retinoic pathway gene expression analyzed in vivo and in vitro in macrophages***.***

**Results:**

We found that administering a single dose of atypical TLR2/4 ligands (CS1 or CS2), 3 days after nitrofen, cured diaphragmatic hernia in 73% of the fetuses and repaired the lesion with complete diaphragm closure being on the other hand nontoxic for the mothers or pups. Moreover, these immunomodulators also improved pulmonary hypoplasia and alveolar maturation and vessel hypertrophy, enhancing pulmonary maturity of fetuses. We also found that CS1 treatment rescued the CDH phenotype in the G2-GATA4^Cre^;Wt1^fl/fl^ CDH genetic mice model. Only 1 out of 11 mutant embryos showed CDH after CS1 administration, whereas CDH prevalence was 70% in untreated mutant embryos. Mechanistically, CS1 stimulated the infiltration of repairing M2 macrophages (CD206^+^ and Arg1^+^) into the damaged diaphragm and reduced T cell infiltration. Additionally, those TLR ligands induced retinol pathway genes, including RBP1, RALDH2, RARα, and RARβ, in the affected lungs and the diaphragm and in macrophages in vitro.

**Conclusions:**

Our research has shown that TLR ligand immunomodulators that influence anti-inflammatory macrophage activation can be effective in treating CDH, being nontoxic for the mothers or pups suggesting that those TLR ligands are a promising solution for CDH leading to orphan drug designation for CS1. The immune system of the fetus would be responsible for repairing the damage and closure of the hernia in the diaphragm and enhanced proper lung development after CS1 treatment.

**Supplementary Information:**

The online version contains supplementary material available at 10.1186/s13023-024-03384-7.

## Introduction

Congenital Diaphragmatic Hernia (CDH) is a rare disease involving a defect in the diaphragm frequently involving pulmonary hypoplasia. It occurs in about 1/3,000–5,000 of newborns (http://www.orpha.net/consor/cgi-bin/Education_Home.php?lng=EN#REPORT_RARE_DISEASES), and accounts for about 50,000 neonatal deaths per year worldwide, and an unknown number of miscarriages. CDH involves a posterolateral diaphragmatic defect that allows herniation of the abdominal organs into the chest and is constantly associated with pulmonary hipoplasia and hypertension. Compression by the herniated abdominal organs further hinder lung development. Severe respiratory distress in the neonatal period occurs in the newborns that still often succumb in spite of treatment (reviewed in [1–4]).

These treatments consist mainly of prenatal reversible tracheal occlusion, neonatal respiratory assistance, pulmonary vasodilators, inotropic agents, surfactant, antibiotics, extracorporeal membrane oxygenation (EMCO), etc. [5, 6] prior to or concurrently with surgical repair of the diaphragmatic defect. The etiology of CDH remains largely unclear and currently is thought to be multifactorial. Multiple genetic factors along with environmental exposures and nutritional deficiencies have been proposed as possible etiologies for CDH. Although 20–40% of cases may have a genetic cause, the remaining ones are idiopathic in origin. Several studies have presented evidence of an increased risk for the development of CDH due to prenatal exposure to several maternal factors, such as alcohol, smoking, low vitamin A intake, obesity and antimicrobial drugs [1].

The etiology of CDH is still poorly known. A widely accepted hypothesis relies on alterations in retinol metabolism leading to deficiencies in retinoic acid (RA) signaling, an important regulator of many genes during embryonic development. Vitamin A is essential for embryonic development and animal models genetically deficient in RA signaling and vitamin A-deficiency are both associated with CDH, which is consistent with the CDH retinoid hypothesis [7, 8]. RA is an important regulator of diaphragm embryogenesis and defects in this pathway, or its downstream targets, can contribute to the development of Bochdalek hernia in CDH [9]. Bochdalek CDH is likely due to a failure of the fusion of pleuroperitoneal folds (PPFs) with the septum transversum (ST) [10]. This fusion may occur with the post hepatic mesenchymal plate (PHMP), an accumulation of mesenchymal cells derived from the ST and located in the posterodorsal margin of the liver lobes [11].

Although there are cases of familial CDH, most genetic data point to a multifactorial inheritance. In fact, multiple genes have been identified in patients with CDH, with COUP-TFII/Nr2f2, Friend of GATA2 (FOG2/zfpm2), GATA4, WT1 and SLIT3 being widely implicated (revised in [12, 13]. Results obtained with genetically deficient animals pointed to the same genes found in patients with CDH, including others implicated in the development and differentiation of the diaphragm and/or lung [12, 13]. On the other hand, there are several animal models that reproduce the symptoms of CDH [14]. These include treatment during gestation with the herbicide teratogen nitrofen [15, 16], being its activity ascribed to alteration in the regulation of RA synthesis [17, 18].

Macrophages are more than just professional phagocytic cells or antigen presenting cells in innate immunity. Their functions also include their homeostatic capacity to repair tissue after injury [19]. Macrophages can be activated in different ways leading to M1 (classical) or M2 (alternative) differentiation [20]. M1 macrophages are considered pro-inflammatory while M2 macrophages are anti-inflammatory and involved in tissue repair. An essential role is increasingly being given to macrophages in tissue remodeling and development during ontogenesis, either directly or through interaction with progenitor cells or stem cells [19, 21]. Large numbers of macrophages are present in almost all developing organs [22]. Importantly, macrophages infiltrate the fetal diaphragm in rats and appeared to be involved in the removal and remodeling of muscle fibers in a homeostatic manner [23]. On the other hand, RA metabolism is a property of M2 macrophages or dendritic cells activated by Toll Like receptor (TLR)2 ligands [24, 25]. Moreover, a recent report has shown that blockade of macrophage inhibitory factor (MIF) results in reduced macrophage migration and ameliorates pulmonary hyperplasia in the nitrofen model of CDH [26].

Macrophages contribute to homeostatic development of the diaphragm and lung, infiltrate and repair damaged muscles [27]. We hypothesized that modulating macrophage migration and activity with specific immunomodulators may remodel damaged diaphragm in CDH. Thus, immunomodulators that can both activate the RA pathway and induce M2 type differentiation repairing macrophages may result beneficial in CDH. RA pathway activation and M2 macrophages would in turn help repair damage caused by teratogens/mutations associated with CDH development and would contribute to tissue remodeling and organ development. To demonstrate this hypothesis, we tested several immunomodulatory candidates (TLR2 ligands), that have those properties in vitro, on CDH animal models and found that TLR2/4 ligands have a significant curative effect.

## Materials and methods

### TLR ligands

Atypical lipopolysaccharides (LPS) from *Rhizobium rhizogenes* K-84 (CS1) [28] and *Ochrobactrum intermedium* (CS2) were purified and characterized as previously described [29]. The purity of those compounds was assessed by mass spectrometry with a purity level higher than 98%. LPS from *E. coli* O111:B4 was from Sigma. TLR2/TLR1 ligand Pam3CSK4 and TLR2/6 ligand FSL-1 were from InvivoGen: All LPS were resuspended in sterile PBS 1x.

### Nitrofen rat model

Female Sprague–Dawley rats weighing 220–250 g and males with proven fertility, were housed in the facilities of the Research Unit of the Hospital Universitario La Paz in Madrid. They were provided with a special diet for rats and had access to water "ad libitum". After controlled fertilization, the time at which the vaginal smear demonstrates the presence of spermatozoa was considered day 0 of gestation [18]. The pregnant female rats were randomly divided in each experiment into two groups: the nitrofen group and the control group. For the nitrofen group 100 mg of nitrofen (2,4-dichloro-4'-nitrodiphenyl ether, Sigma-Aldrich) in 1 mL of olive oil was administered via intragastric on day 9.5 of gestation [16]. The untreated control group received an equivalent volume of olive oil (placebo) using the same administration method. Subsequently, the nitrofen group was further divided into two subgroups: one subgroup was intraperitoneally injected with the immunomodulators added at different times after nitrofen (where indicated), at a single dose of 100 µg/Kg. The other subgroup received an equal volume of saline, serving as the nitrofen untreated group. On gestational day 18 or 21, the pregnant rats were euthanized by intracardiac injection of potassium chloride, and all the fetuses were carefully collected by caesarean section. The fetuses' diaphragms were examined for the presence of hernias under a stereomicroscope. Then, they were categorized into five groups for subsequent analysis, based first in the treatment they received olive oil or Nitrofen, and then if they were treated with immunomodulator or just with phosphate buffered saline (PBS). In addition, fetuses from the nitrofen-treated mothers’ groups were further subdivided, in those having or nor hernias since the nitrofen treatment of mothers not always resulted in the presence of hernias in all their fetuses. In summary, the 5 groups were the placebo (healthy) control group and four nitrofen-treated groups: nitrofen + saline with hernia group (Nitro CDH +) nitrofen + saline without hernia group (Nitro CDH-), nitrofen with immunomodulator treatment but still presenting hernia group (Nitro + Immunomodulator CDH +), and nitrofen with immunomodulator treatment but no hernia group (Nitro + Immunomodulator CDH-). From the various subsequent analyses, n = 3 fetuses, from 2 different mothers, in the control group and n = 5 fetuses, from 2–3 different mothers, in the all of the nitrofen groups were analyzed per experiment.

Throughout the process, the animal experimentation followed ARRIVE guidelines and received approval from the Fundación para la Investigación Biomédica del Hospital Universitario La Paz ethics committee. The protocols were in accordance with Spanish legislation (RD 53/2013) and European legislation (2010/63/EU).

### WT1 conditional KO (G2-GATA4^Cre^;Wt1^fl/fl^) mice

The G2-GATA4^Cre^;Wt1^fl/fl^ mice model for CDH was previously described [10]. In short, we used a driver based on the G2 enhancer of the Gata4 gene that drives expression of Gata4 in the lateral plate mesoderm from the stage E7.5, and by the stage E9.5 is active in the septum transversum (ST) and proepicardium, ceasing its activity by E12.5. The activity of this enhancer is completely absent in the intermediate mesoderm. Those mice were crossed with the Wt1 floxed mice *WT1*^*fl/fl*^ which allow the expression of the Cre recombinase and subsequent deletion of WT1 only on those tissues and in those days. Defect in the PHMP in G2-Gata4^Cre^;Wt1^fl/fl^ mutant embryos could be observed as early as E10.5. It provides a good model on the genesis of the Bochdalek hernia [10].

Pregnant females at E9.5 and E10.5 were administered a single intraperitoneal injection (IP) of CS1 diluted in PBS at a dose of 100 µg/Kg of animal weight. Embryos were isolated at E15.5. Whole mount embryos were fixed in 4% paraformaldehyde in phosphate-buffered saline (PBS) at 4 °C during 4–5 h and processed for paraffin embedding. Hematoxylin–Eosin stain was performed using routine protocols. All embryos were staged from the time point of vaginal plug observation, which was designated as E0.5. Embryos were excised and washed in PBS before further processing.

The procedures used in this study were in compliance with the institutional and European Union guidelines for animal care and welfare and approved by the Committee on the Ethics of Animal Experiments of the University of Malaga (permit code 2015–0028).

### Isolation of mouse peritoneal macrophages

C57BL/6 WT, TLR2 and TLR4 KO mice littermates were obtained from S. Akira. All mice were bred and maintained in the animal facilities of the Centro de Biología Molecular Severo Ochoa in Universidad Autónoma de Madrid. All animal procedures were performed in strict accordance with the European Commission legislation for the protection of animal used purposes (2010/63/EU). The protocol for the treatment of the animals was approved by the Comité de Ética de la Dirección General del Medio Ambiente de la Comunidad de Madrid, Spain (permits PROEX 128/15). Thioglycolate-elicited peritoneal macrophages were isolated from 6–8-week-old pathogen-free mice. Cells were cultured in RPMI 1640 (2 mM L-glutamine, antibiotics 100 units/mL penicillin, 100 mg/mL streptomycin) with 5% fetal bovine serum (FBS) and seeded into 6-well-pates at a density of 1 × 10^6^ cells/well. Cells were allowed to adhere for 2 h and then the medium was changed to remove non-adherent cells. After 24 h, medium was replaced with new complete medium prior treatment with TLR ligands [29].

### Morphometric and immunohistochemistry studies

Morphometric analyzes of fetuses, lungs or diaphragm were performed on E18 and E21 slides, stained with Hematoxylin/Eosin as described [30]. For lung morphometric studies, we analyzed the ratio of empty space to cellular space in lungs as a surrogate of alveolar space. This was analyzed from 4 different regions of 4 different fetuses in each group. Radial alveolar counts were performed analyzing several images of 4 fetuses from each group as described [31]. Alveolar thickness was measured as the mean septal wall thickness of terminal alveoli per field. Elastin staining was used to study the morphometry of the vessels using Elastica Van Gieson Kit (Merck) following manufactured condition. Measurements were made of the aorta artery in E18 thoracic sections in 3 fetuses from each group. Increase of the wall thickness to vessel radius ratio (w/r) is related with increasing systemic pressure [32].

The expression of immune system markers was mostly studied with inmunohistochemistry using the following antibodies: mouse anti rat CD68 (dilution 1:300 BIORAD, ref. MCA 341R) for monocytes/macrophages, rabbit anti CD3 (dilution, 1:400; Biorbvt, ref. orb10313) for lymphocytes T, rabbit anti CD20 (dilution, 1:400; Bioss, ref. bs-0080R) for lymphocytes B, rabbit anti mouse CD206 (dilution, 1:200; Bio Orbyt, orb180464) and rabbit anti human p67 (dilution 1:1000, Bioss antibodies). As secondary antibodies, Anti-Rabbit horse rabbit peroxidase (HRP) (Millipore) for CD20 and CD3 and Anti-mouse HRP (Millipore) for CD68.

The samples were deparaffinized and pretreated to pH6 using PT-link (Dako-Agilent). Subsequently, they were incubated for 30 min hydrogen peroxide solution, to block the activity of endogenous peroxidase, and after that they were incubated for one hour with blocking solution (Tris-Buffered Saline (TBS) + 10% normal goat serum + 1% bovine serum albumin + 0, 01% Triton X-100) to block non-specific binding of antibodies. They were then incubated with the corresponding primary antibody at 4 °C overnight. Incubation with the secondary antibodies, was performed at room temperature for 45 min followed by three 5-min washes in TBS. The signal was revealed with 3,3'-Diaminobenzidine (DAB) substrate (Palex) and the nuclei were counterstained with Mayer hematoxylin (Merck). The incubations were carried out in humid chambers to prevent the samples from drying out. Once the staining process is finished, the slides were dehydrated in an increasing alcohol gradient and finally the samples were mounted using DPX (Merck). In order to separate non-specific from specific labeling in the immunohistochemical tests, negative controls were performed, in which the primary antibody was not included and therefore any labeling observed was considered non-specific.

The images were obtained using the Microscope Olympus BX41 and the software ImagePro plus 5.3 and analyzed with Image J program. For immunohistochemistry evaluation, three different areas of each lung and five different parts of the diaphragms were chosen for analysis in 5 fetus of each group and a “positive cell number/total cell number ratio” was stablished.

Confocal immunofluorescence was performed basically as described [33]. Diaphragms, 3 fetuses per group, were fixed in 4% paraformaldehyde in PBS solution, incubated in 30% sucrose solution, embedded in Tissue-Tek O.C.T. compound (Sakura), and frozen in liquid nitrogen. Sections that were 10- to 15-µm thick were fixed in acetone. Incubation with the following antibodies was done at 4 °C: 10 µg/mL goat anti-mouse CD206. Images were obtained using an LSM510 Meta confocal laser coupled to an Axiovert 200 (Zeiss). Four different parts of the diaphragms were chosen for analysis.

### mRNA isolation and quantitative reverse transcription polymerase chain reaction (RT-qpcr)

Some of the lungs, spleens and diaphragms were separated and frozen at -80ºC for RT-PCR which was basically performed as described [34]. Briefly, total RNA was extracted from each organ extracted, previously frozen at -70⁰C, using NZyol Reagent (NZYTech). cDNA was prepared by reverse transcription (GoTaq 2-Step RT-qPCR System, Promega) and amplified by PCR using SYBR® Green PCR Master Mix and ABI Prism7900HT sequence detection system (Applied Biosystems), with the primers shown in Additional file 1: Table S1. The 2 − DDCt method was applied to analyze the relative changes in expression profiling and all quantifications were normalized to the housekeeping gene RPL13A.

For mouse peritoneal macrophages, cDNA was prepared by reverse transcription (GoTaq 2-Step RT-qPCR System, Promega) and amplified by PCR using SYBR® Green PCR Master Mix and ABI Prism 7900HT sequence detection system (Applied Biosystems). Primers used for qPCR analysis are listed in Additional file 1: Table S2.

### Statistical analysis

One way ANOVA was carried out, with a Bonferroni’s post-test for multiple Analysis was performed using GraphPad Prism 5 software. Quantitative results are expressed as means ± SEM or mean ± SD. A p value less than 0.05 was considered statistically significant.

## Results

### Effect of TLR ligands in nitrofen-induced CDH

**Fig. 1:**
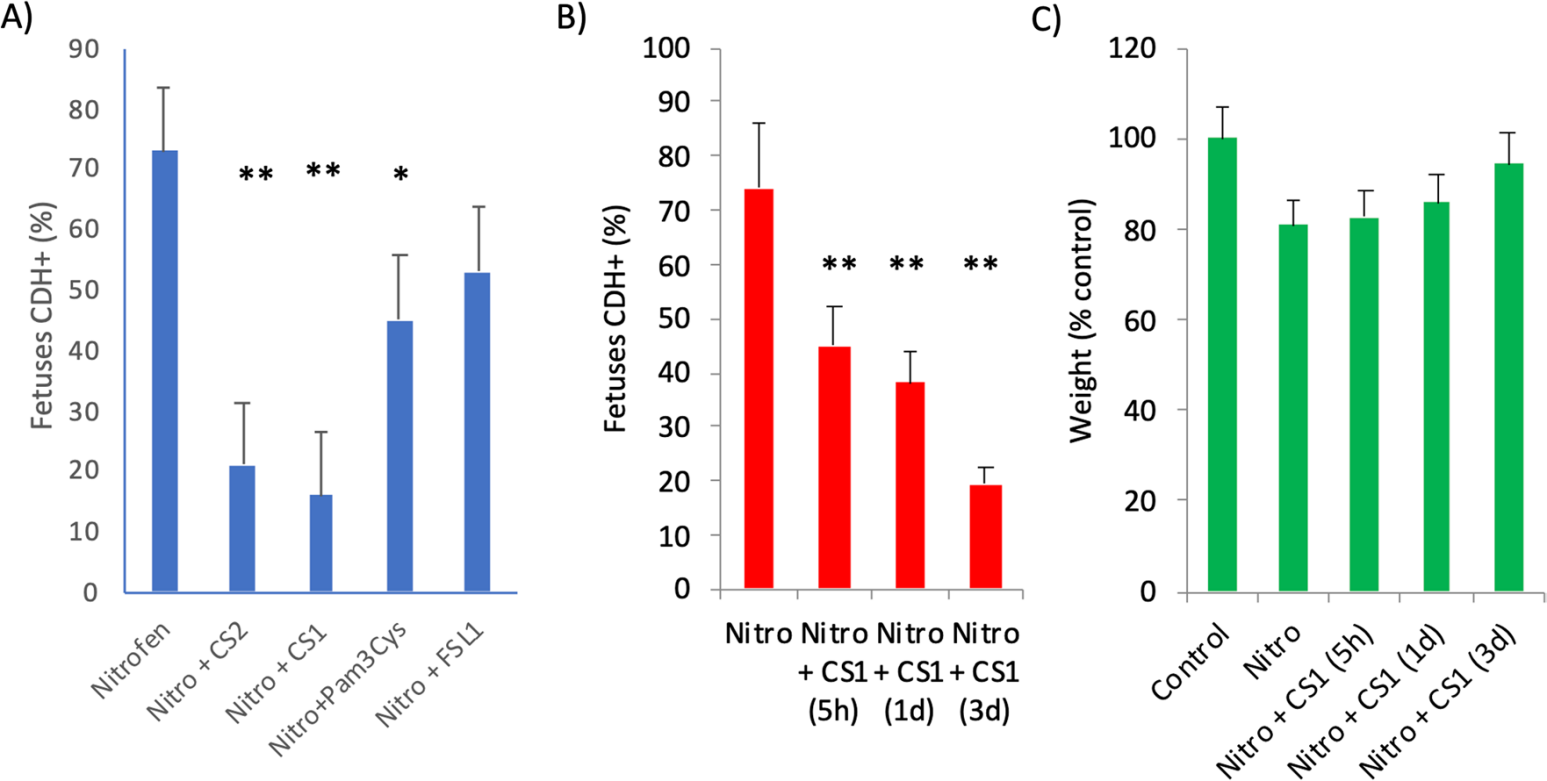
Effect of TLR ligands in diaphragmatic hernia induced by nitrofen.** A** Pregnant Wistar rat females were administered nitrofen (n = 6), or olive oil as placebo (control) (n = 2), at E9.5 and 3 days later injected intraperitoneally with 100 μg/Kg of the indicated immunomodulators or only saline (None or Untreated). Results are represented as percentages of all fetuses pregnant rats with hernia (CDH +) at E21.** B** and** C** Pregnant Wistar rat females were administered nitrofen at E9.5 and 5 h, 1 day or 3 days later injected intraperitoneally with 100 μg/Kg of CS1 or saline.** B** Diaphragmatic hernia percentage. Results are represented as percentages of all fetuses with hernia (CDH +) at E21.** C** The weight of the fetuses recorded at E21. **p > 0.001, *p > 0.01 respect to untreated nitrofen group (control). In this case, all fetuses from 3 rats from each group, independent or not of the presence of hernia, were analyzed

Initially, we tested several TLR2 ligands in a rat model of CDH induced by nitrofen. Nitrofen was administered to pregnant rats at embryonic stage E9.5 to induce CDH and 3 days later (E12.5), in order to allow organ alterations to appear, several TLR ligands were injected. On day 21st of gestation, the rats were sacrificed and the embryos were examined for the presence or absence of diaphragmatic hernia (CDH + or CDH-, respectively). Pregnant rats exposed to the herbicide nitrofen had between 60–85% of fetuses with CDH (73% on average in Fig. 1A) in line with many previous studies [35]. In this model, the severity is high and hernia is prominent. Thus, we found that all herniated fetuses have left involvement with liver in the thorax and 75% both left and right side in agreement to what it has been described [36]. Treatment of the pregnant mothers with a single dose of TLR2/1 or TLR2/6 ligands, 3 days after nitrofen administration reduced on average CDH + fetuses from 73% in saline only treated animals to 46% and 52% respectively (Fig. 1A). This represents a relative decrease (or improvement) in CDH incidence caused by nitrofen of 37% and 29% respectively. More importantly, atypical LPS which are a new class of dual TLR2-TLR4 ligands from soil bacteria (rhizobiales) [29] were much better at reducing this CDH incidence. Thus, the LPS from *Rhizobium rhizogenes* K-84 (from now on referred as CS1) reduced CDH incidence to only 16% whereas LPS from *Ochrobactrum intermedium* (referred as CS2) resulted in a 21% incidence. This represents a relative decrease of 79% and 72% in hernia incidence respect to the nitrofen with saline treatment (Fig. 1A). Treatment with LPS from *E.coli* was toxic to the fetuses, and no fetuses were recovered.

Full severe hernia may take some time to appear after nitrofen. Thus, adding the compounds at different times aftrer nitrofen, may distinguish “preventive” from “curative” effects of compounds. We treated with CS1, 5 h, 1 or 3 days after nitrofen exposure at day E9.5. Interestingly, the efficiency in reducing the percentage of herniated CDH + fetuses was higher when the treatment with CS1 was applied 1 day and especially 3 days after nitrofen administration, reaching then the maximum healing of the hernia (Fig. 1B). Average embryo weight showed a slight increase following treatment with CS1 compared to saline-treated rats, (Fig. 1C). Although, both CS1 and CS2 compounds behaved very similarly in all biological assays, only those for the best one (CS1) are shown below.

**Fig. 2:**
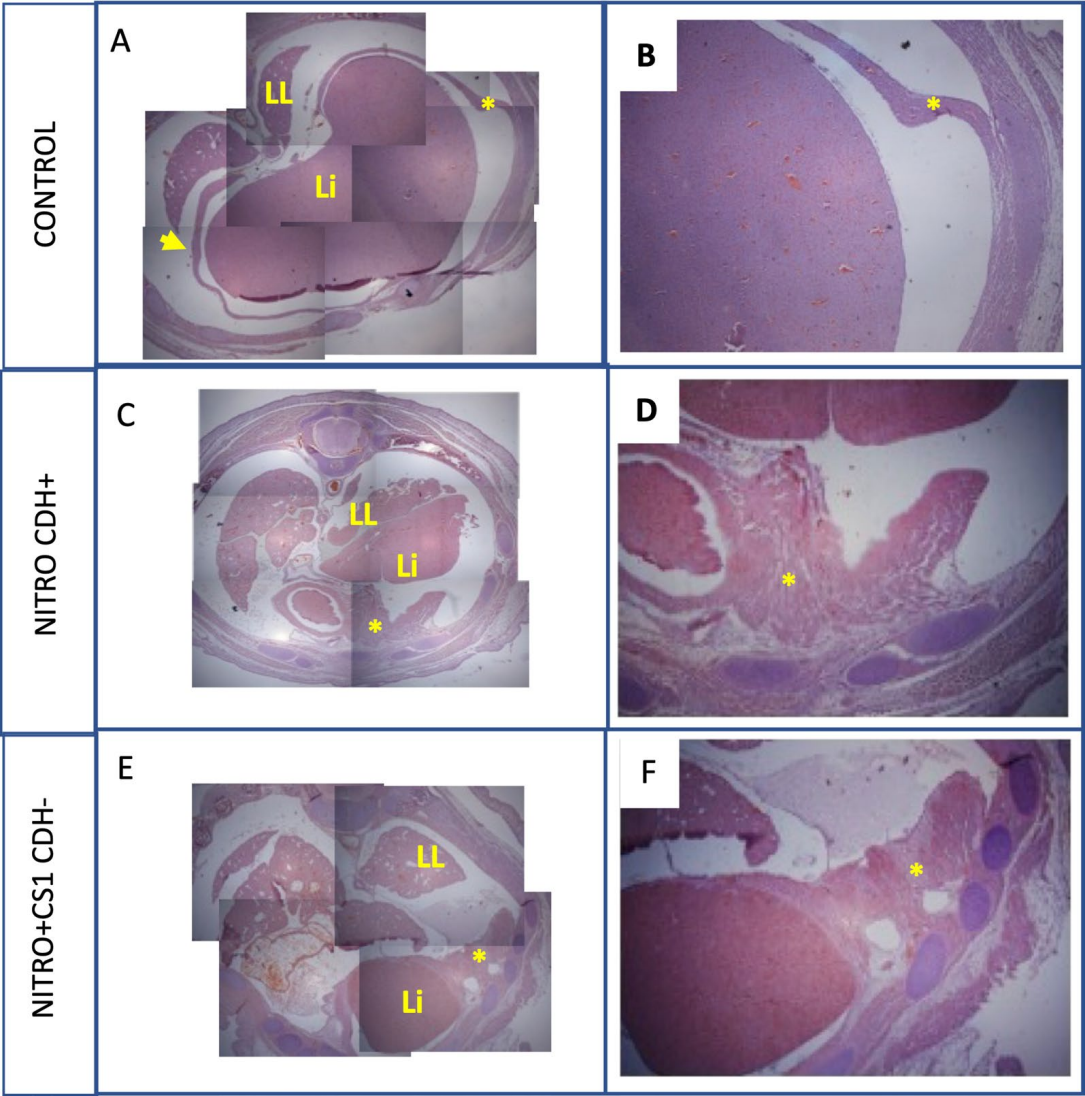
Effect of CS1 on the diaphragms of nitrofen-treated fetuses. Pregnant Wistar rat females were administered nitrofen, or olive oil (control), at E9.5 and 3 days later injected intraperitoneally with 100 µg/Kg of CS1 immunomodulator or only saline. Histological analyses of the fetuses were performed at E21. The representative images show fetuses from the healthy (Control), nitrofen with hernia (Nitro CDH +) and CS1-treated nitrofen without hernia (Nitro + CS1 CDH-) groups. (**A**, **C**, **E**) photographic composition of a cross-section of the fetuses of the different groups. A complete diaphragm is clearly observed in the control group while in the fetuses treated with nitrofen the liver (Li) has invaded the thorax due to the absence of the diaphragm. In the nitrofen fetus treated with the CS1 a complete diaphragm is also observed. (**B**, **D**, **F**) magnification of the diaphragm (*). The control fetus diaphragm (**B**) shows a normal morphology and homogeneous muscle fibres separating the abdomen from the thorax (**A**, arrow). In contrast, the incomplete diaphragm of the (Nitro CDH +) group (**D**) presents an amorphous morphology. Although there was no hernia in Nitro + CS1 CDH- group disorganised muscle fibre structure was also observed (**F**)

The histological analysis of fetuses from pregnant mothers treated with CS1 were analyzed at E21 (Fig. 2). In healthy fetuses, a complete diaphragm is clearly observed with a normal morphology and homogeneous muscle fibers separating the abdomen from the thorax. As expected, fetuses from mothers that were administered nitrofen presented with an invasion of the thorax by the liver, due to partial or total absence of the diaphragm, combined with amorphous morphology. Importantly, treatment with CS1 immunomodulator prevents damage to the diaphragm. Thus, at E21, most of fetuses of nitrofen administered mothers and treated with the immunomodulator, presented a complete diaphragm although with a more disorganized and hypertrophied morphology (Fig. 2 D,E,F).

Alteration and retarded development of the lung is a characteristic of CDH [1, 3]. Histological analysis of the embryonic lungs showed clear differences among the groups treated with CS1 at E18 or E21 (Additional file 1: Fig. S1). Those lungs from nitrofen CDH + fetuses were notably smaller than the rest including the CS1-treated CDH + fetuses (with herniation), and even smaller than CS1-treated CDH- fetuses (without herniation). Furthermore, the lungs of CDH + nitrofen fetuses showed altered morphology with less defined lobes. Importantly, CS1-treated nitrofen fetuses exhibited lung morphology resembling that of normal mature lungs in 21-day-old fetuses with an increased number of respiratory bronchioles, alveolar ducts and alveoli (Additional file 1: Fig. S1), while saline treated CDH + fetuses displayed a more immature lung appearance, with underdeveloped and less clearly defined alveolar structures. The differences in alveolar ducts, alveoli, and respiratory bronchioles were evident as well. The untreated nitrofen group showed reduced airspace, especially in CDH + fetuses, when compared to the treatment group and healthy controls. In contrast, the treatment group demonstrated better lung development in terms of size and an increased number of alveolar ducts especially in CDH- fetuses.

**Fig. 3:**
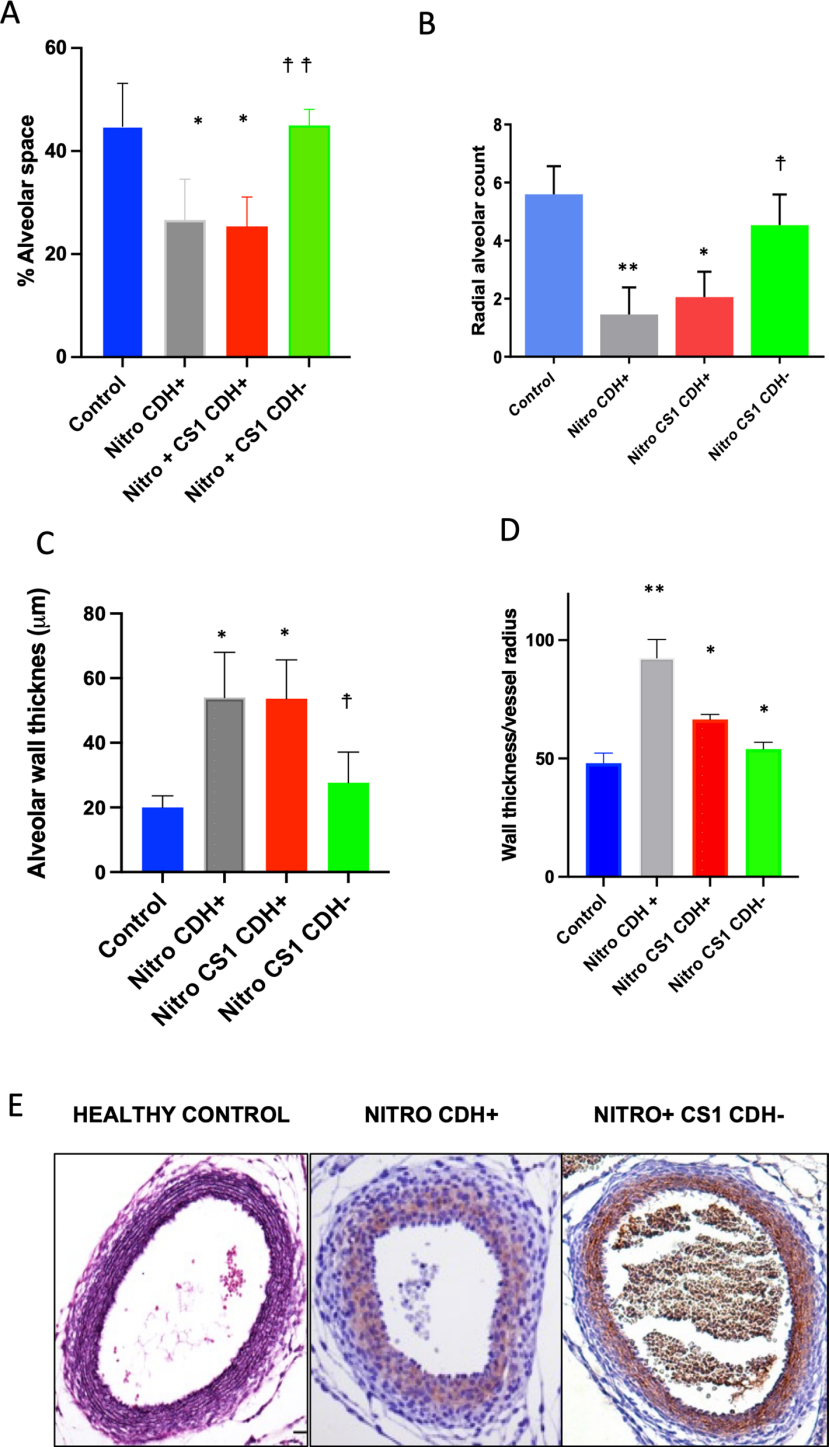
Effect of TLR ligand in lung development. Pregnant Wistar rat females were treated with nitrofen at E9.5 and 3 days later injected intraperitoneally with 100 µg/Kg CS1. **A** Percentage of empty alveolar to cellular space in lungs. This was analyzed from 4 different regions of 4 different fetuses in each group. **B** Radial alveolar counts were performed analyzing several images of 4 fetuses from each group as escribed in methods. **C** alveolar thickness was measured as the mean septal wall thickness of terminal alveoli per field of the field measured. **D** Wall thickness/vessel radius ratio. Analysis of E18 lung vessels from the different groups. 3 independent calibrations for different lung sections of 3 different fetuses were staining with van Giemson. **E** Representative van Giemson staining of the indicated groups:. **p > 0.001 and *p > 0.01, respect to control healthy group; ☨☨p > 0.001 and ☨p > 0.01 respect to untreated CDH + nitrofen group

Quantification of the alveolar volume indicates that it was reduced around 40% in CDH + nitrofen fetuses, whereas was similar to control placebo olive oil control rats in nitrofen CS1-treated CDH- fetuses (Fig. 3A). Morphometric analyses were conducted to assess pulmonary vascular hypertrophy. Quantification of radial alveolar count demonstrated a substantial reduction in animals CDH + after nitrofen treatment that was greatly substantially recovered in nitrofen CS1-treated CDH (Fig. 3B). In contrast, alveolar wall thickness increased after nitrofen and again was reversed in CS1-treated CDH- animals (Fig. 3C).

Healthy controls exhibited a highly stained media layer of the aorta wall with Van Giemson staining. In contrast, the nitrofen-CDH + showed reduced staining of elastic fibers, indicating areas of tissue injury (Fig. 3D-E). However, CS1 immunomodulatory treatment reversed this effect, supporting the beneficial impact of CS1 treatment in mitigating the negative effects of pulmonary hypoplasia and associated pulmonary vascular changes in CDH.

### Effect of TLR ligands in WT1 conditional KO (G2-GATA4^Cre^;Wt1^fl/fl^) mice

**Fig. 4:**
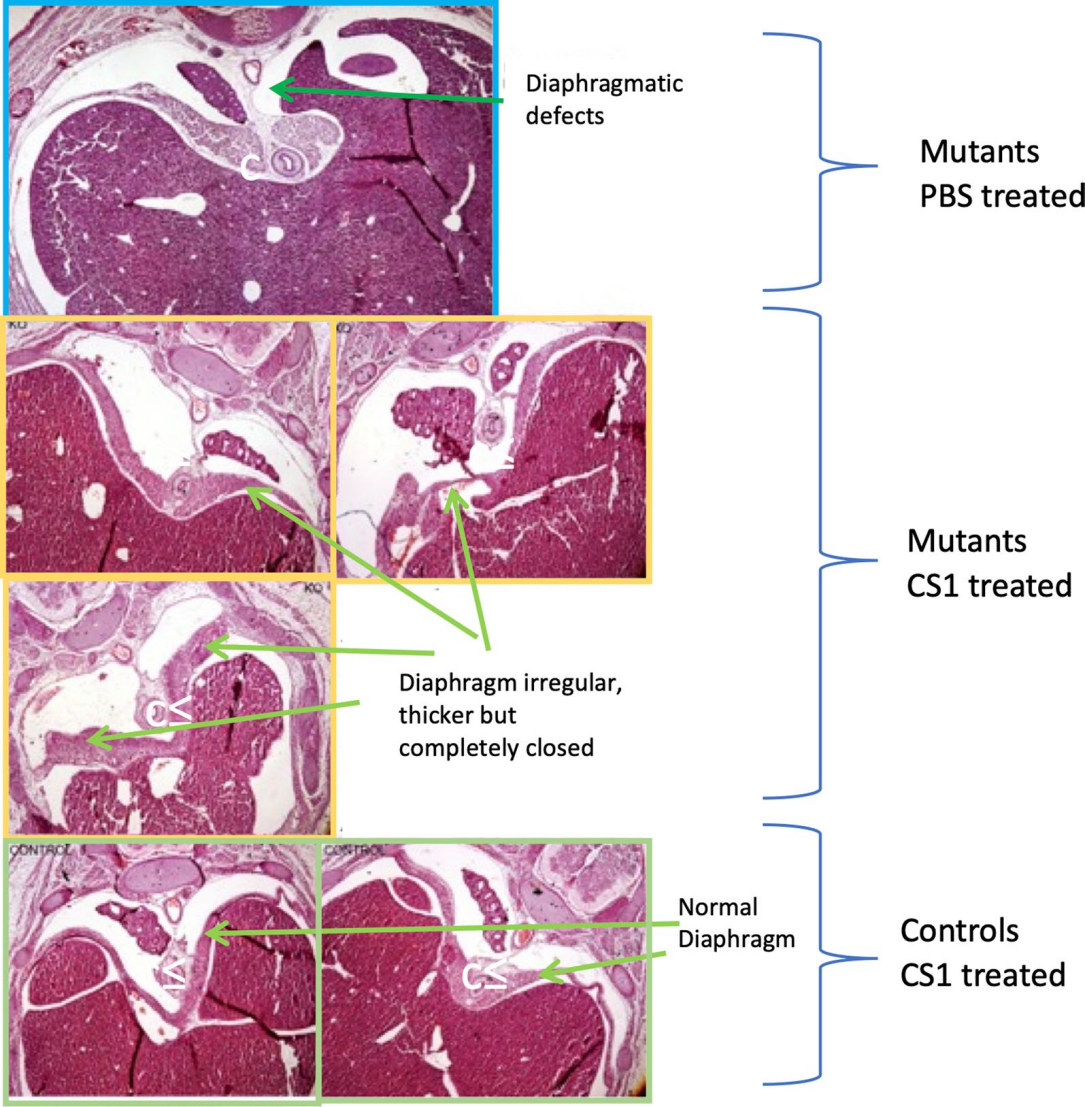
Effect of CS1 in G2-GATA4Cre;Wt1fl/fl mice. Pregnant mice mothers were treated with 100 µg/Kg of CS1 or saline intraperitoneally, twice at E9.5 and E10.5. Embryos were analysed at E15.5. Images representatives from WT1 mutant embryo of mothers treated with PBS or CS1. Embryos from control not mutant mothers are also shown

Next, we decided to confirm the effects of CS1 treatment, using a genetic model, the G2-GATA4^Cre^;Wt1^fl/fl^ mice, with conditional ablation of the Wilms’ tumor suppressor gene (*Wt1*) in lateral plate cells expressing GATA4 under the control of the G2 enhancer [10]. The use of this WT1 conditional knockout overcomes the early embryonic death caused by systemic deficiency of WT1 and it constitutes a valuable animal model of CDH. In this model, WT1 is involved in the generation of the mesenchyme of the ST/PHMP/PPFs continuum through epithelial-mesenchymal transition. Defect in the PHMP in G2-Gata4^Cre^;Wt1^fl/fl^ mutant embryos and a strong reduction of the ST mesenchyme could be observed as early as E10.5 G2-Gata4^Cre^;Wt1^fl/fl^ mutant embryos as previously described [10]. Thus, we treated G2-GATA4^Cre^;Wt1^fl/fl^ pregnant mice mothers with CS1 or PBS intraperitoneally twice at E9.5 and E10.5 about 2 days later than the WT1 deletion in the embryos was genetically induced. Embryos were analyzed at E15.5 (Fig. 4 and Additional file 1: Fig. S2). In absence of treatment about 70% of the embryos developed typical Bochdalek-type CDH, with diaphragmatic defect, liver invasion of the left pleural cavity and hypoplasia of the left lung. Very remarkably, CS1 treatment rescued the CDH phenotype in the G2-GATA4^Cre^;Wt1^fl/fl^ model. Of the 11 mutant fetuses analyzed, only 1 had diaphragmatic hernia (only 9% incidence) (Fig. 4). In all but one CS1-treated mutants the pleural cavities were completely closed in contrast to the untreated mutant mice (Additional file 1: Fig. S2). Very importantly, the sinus venosus of all CS1-treated embryos was abnormal indicating that WT1 was in fact deleted in all CS1-treated animals (Additional file 1: Fig. S2). This is an internal control to confirm the deletion of WT1 and indicates that hernia was healed by CS1 and that the observed decrease on CDH was not due to a deficient deletion of the WT1 gene in those animals treated with CS1. Although the diaphragm in most cases does not appear normal (as in immunomodulator-treated nitrofen CDH + rats), being more irregular, with less organized musculature and hypertrophied, it was completely closed (Additional file 1: Fig. S3). It is also important to mention that control embryos treated with CS1 were normal (Fig. 4 and Additional file 1: Fig. S3). Treatment with CS1 was neither toxic to the normal pregnant mothers.

### Immune cell infiltration in damaged organs in CDH

**Fig. 5:**
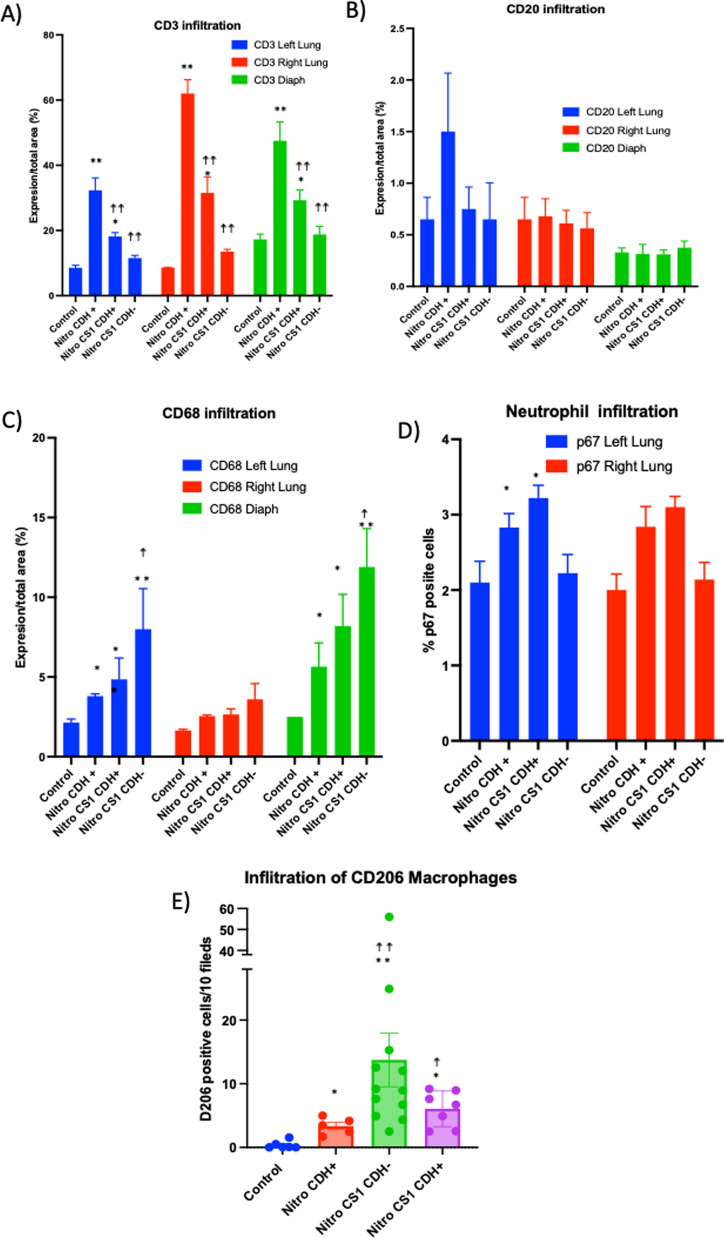
Immune infiltration in lung and diaphragm induced by nitrofen and CS1 treatments. Pregnant Wistar rat females (3 per group) were treated with nitrofen or olive oil (control) at E9.5 and 3 days later injected intraperitoneally or with 100 µg/Kg CS1 or saline. **A**–**D** Indicated cell infiltration in the diaphragm, left and right lung was determined by immunohistochemistry at E21. In the CS1 treated groups, both CDH-negative and CDH-positive fetuses were analysed separately. **E** CD206 expression was determined by immunofluorescence at E18. **p > 0.001 and *p > 0.01, respect to control normal group; ☨☨p > 0.001 and ☨p > 0.01 respect to untreated CDH + nitrofen group

First, we measured immune cell infiltration through the expression markers of macrophages (CD68 +), B (CD20 +) and T (CD3 +) lymphocytes and neutrophils (p67 +) with immunohistochemistry techniques (Fig. 5). In the CS1 treated groups, both CDH- and CDH + fetuses were analyzed separately.

In rats with CDH induced by nitrofen, a significant and high increase in CD3 T cell infiltration was observed in both the left and right lungs as well as in the diaphragm of the embryos at E21. However, in fetuses from pregnant rats treated with CS1, this infiltration was notably reduced, even in the few cases where the diaphragm did not close completely. This reduction was particularly remarkable in the “cured” CDH- fetuses (Fig. 5). No relevant effect was observed in B cell infiltration (Fig. 5). Examining the percentage of p67 + cells, a marker for neutrophils, in lung tissue, we found a lower number of p67 + cells in both the control and CS1-treated CDH- groups (Fig. 5). This indicates a higher neutrophil infiltration in CDH + pups. No p67 + infiltrating cells were detected in the diaphragms.

We observed a significant increase of macrophage infiltration (CD68 + cells in all CDH + groups compared to the control or CDH- groups. Interestingly, although nitrofen induced an infiltration of CD68 + macrophages in left lung and diaphragm, CS1 significantly increased this infiltration, especially in the CDH- fetuses (Fig. 5). To corroborate this, we specifically studied the repairing macrophage population by using the M2 macrophage specific marker CD206 (Fig. 5E). A great infiltration by M2 macrophages was observed in all nitrofen-treated animals. However, in CS1-treated CDH- animals the infiltration of this M2 population to the diaphragm was 2–threefold higher than in nitrofen CDH + animals. This indicates that there is an active recruitment of these M2 cells to facilitate the repair of the damaged tissue in CS1-treated CDH- animals. Infiltration in the lungs and diaphragm in the CS1-treated but still with hernia CDH + , animals is modified with the same tendency than Nitro + CS1 CDH- animals, although in almost all cases (except for CD206 macrophages and CD3 T cells) did not reach statistical significance.

### Gene expression regulation by CS1 in CDH

**Fig. 6:**
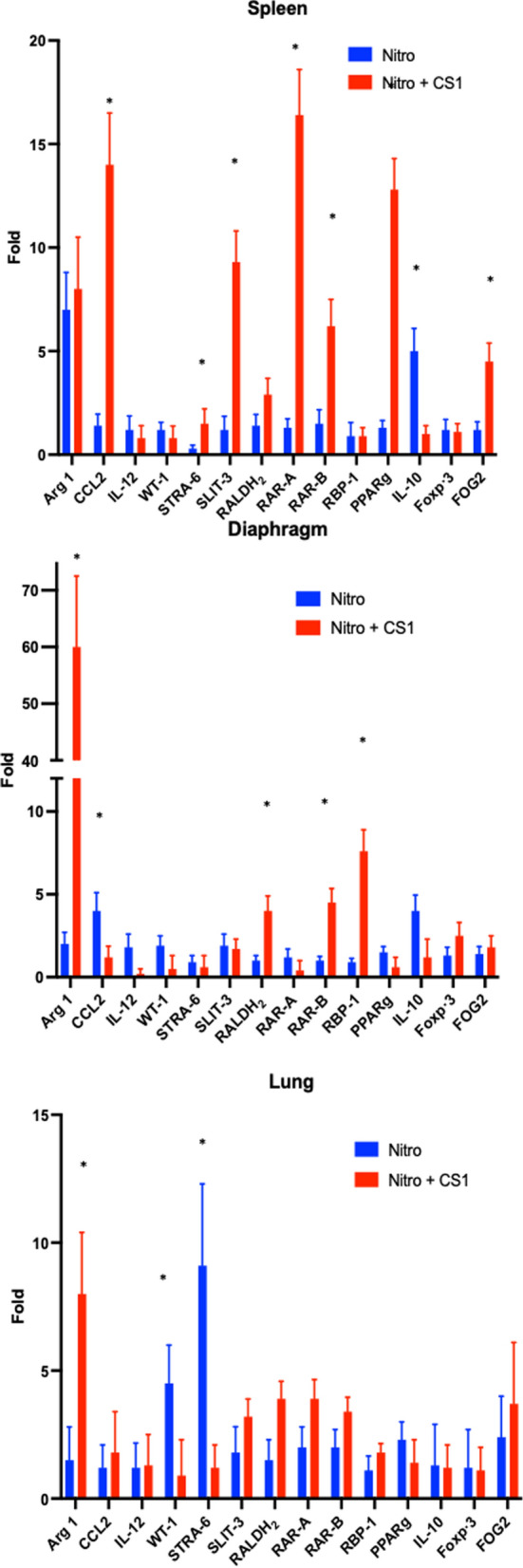
Effect of TLR ligand CS1 on gene expression in vivo. Pregnant Wistar rat females were treated with nitrofen or olive oil (placebo) at E9.5 and 3 days later injected intraperitoneally with 100 µg/Kg CS1 or saline. RT-PCR of mRNAs of the indicated proteins in the Diaphragm, Spleen or Lung organs was carried out at E21. Results are the mean of 2 independent experiments with 5 fetuses per group each. Results are shown normalized to controls of untreated rats. *p > 0.01, respect to untreated CDH + nitrofen group

To gain a better understanding of the effects of CS1 in tissues, we analyzed the gene expression of immune, and developmental or RA pathway genes, known to be related to CDH [12, 13], in the lungs, diaphragm, and spleen. Figure 6 provides a summary of two independent experiments, each including all fetuses from pregnant rats treated with nitrofen at E9.5, independent of the presence or absence of hernia. The results were normalized for comparison with the untreated control group using real time QC-PCR. Fetuses from control animals treated with CS1 at E12.5 showed no significant changes in gene expression at E21 compared to fetuses from untreated control rats. Nitrofen administration alone induced few changes in gene expression in the analyzed organs at E21. The most notable ones were the induction of *Arg1* in the spleen and diaphragm, *Ccl2* in the diaphragm, and *Wt1* and *Stra6* in the lungs. Interestingly, treatment with CS1 of nitrofen animals resulted in a significant increase in *Arg1*, *Aldh1a2*, *Rbp1* and *Rarb* in the diaphragm. The spleens from fetuses of CS1-treated rats also showed increases in *Arg1*, *Aldh1a2*, *Rara*, *Ccl2*, *Pparg*, and *Slit3*. In the lungs, however, only *Arg1* showed a significant and high induction following CS1 treatment. Additionally, there was a tendency for increased expression in retinoic pathway genes *Aldh1a2, Rara* and *Rarb*, while the inductions of *Stra6* and *Wt1* caused by nitrofen were reduced by CS1. These results indicate a significant increase in Arg1 and the RA pathway due to CS1 treatment in the affected organs and spleen.

### CS1 induce retinoic pathway genes in macrophages

**Fig. 7:**
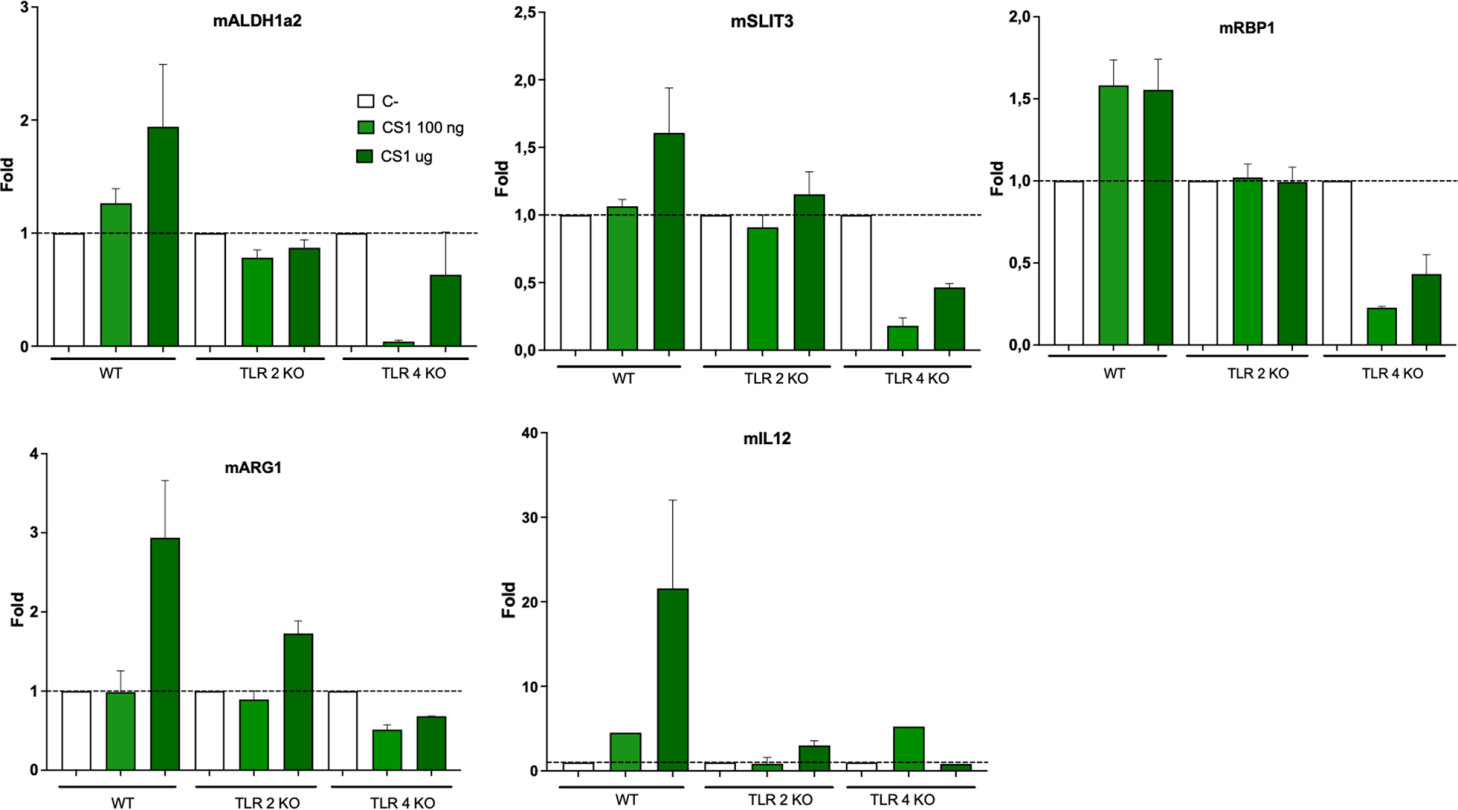
Effect of TLR ligands on gene expression by macrophages. Peritoneal macrophages from WT, TLR2 or TLR4 deficient mice were stimulated with CS1 at the indicated doses and 14 h later the gene expression *Aldh1a2, Slit3, Rbp1, Arg1* and *Il12* was analysed by RT-PCR. (*) p > 0.001

To further understand the effect of the treatments in the immune activation, we investigated the ability of CS1 to activate macrophages in vitro (Fig. 7). CS1 induced RA genes *Aldh1a2* and *Rbp1*, *Arg1,* a marker of M2 macrophages and also weakly induced *Slit3* which has recently been found to be also expressed in M2 mouse peritoneal macrophages [37]. In addition to those genes, CS1 also induced *Il12*. Interestingly, by using macrophages from TLR2 or TLR4 deficient macrophages, we found that those effects were dependent on both TLR2 and TLR4 receptors. These in vitro effects align with the observed infiltration of macrophages and gene expression profile primarily observed in the diaphragm, but also in spleen and lungs, of the CS1-treated fetuses shown above.

## Discussion

In this study, we demonstrate the efficacy of a new class of anti-inflammatory immunomodulatory TLR ligands, atypical LPS, for the treatment of CDH, in both, teratogen-induced and genetic mouse preclinical models. Notably, we observed successful CDH rescue in a genetic model of CDH, the WT1-deficient mice with ablation of Wt1 in the territories giving rise to the diaphragm, making it the first instance, to our knowledge, of immune modulation rescuing the consequences of an inborn genetic defect. Our findings show that the CS1, which modulate TLR2/4, can effectively redirect the retinoic acid pathway and the innate immune system in CDH promoting tissue repair and remodeling. CS1 induces the infiltration of M2 macrophages, particularly in the diaphragm, and significantly reduce the number of hernias in the fetuses of pregnant females after CDH induction with nitrofen. Moreover, CS1 treatment also improves pulmonary hypoplasia and vessel hypertrophy, enhancing pulmonary maturity of fetuses. Additionally, CS1 treatment induces the expression of retinoic acid pathway genes in damaged organs.

Very little is known about those LPS from rhizobial bacteria. They are considered atypical since to difference to LPS from pathogenic gram- bacteria, they have an unusual lipid A with a very long fatty acid chain esterifying the 2 glucosamines [38] that alter their TLR recognition [39]. This results in being able to interact with TLR2/TLR4 heterodimers and becoming anti-inflammatory [29].

Macrophages can be activated in response to different TLR ligands and undergo M1 (classical) or M2 (alternative) activation [20]. M1 is implicated in inflammation and pathogen elimination, and M2 in reparation, tissue remodeling, and wound healing. Arginase I (Arg1) is predominantly expressed in myeloid cells being together with CD206 characteristic markers of M2 macrophages. Interestingly, in CDH fetuses we found higher levels of Arg1 in damaged organs and greater infiltration of CD206 + cells in the diaphragm after CS1 treatment suggesting that CS1 induces infiltration of M2 macrophages in the damaged organs.

Treatment with CS1 could result in a dual action beneficial for prenatal treatment of CDH. Firstly, it can activate repairing macrophages favoring their migration to the damaged organs where they will mitigate tissue damage while favoring remodeling. This concept aligns with studies showing that blockade of MIF, a cytokine capable of inhibiting macrophage migration favors infiltration by these cells and improves pulmonary hyperplasia in CDH models [26]. Secondly, immunomodulators promote in situ synthesis of RA pathway molecules such as RALDH2 which will increase RA levels favoring appropriate organ development. Combining these two actions, promoting repairing macrophages and increasing RA levels, could potentially enhance the overall prenatal therapeutic effect for CDH.

During ontogenesis, macrophages, which are present in almost all developing organs [22], interact with progenitor cells or stem cells contributing to tissue repair and remodeling [19, 21]. An important function of macrophages in ontogenetic development is their trophic role since providing key trophic factors for the development of all tissues [19]. Macrophages are also required to eliminate apoptotic cells produced during normal development of various tissues [40]. It has been described that macrophages infiltrate the fetal diaphragm in rats [23]. These macrophages are frequently observed in the diaphragm during gestation (from day 16) and up to 2–4 weeks of life. During this period, they seem to be involved in the removal and remodeling of muscle fibers in a homeostatic manner. Those evidences fit with our infiltration data in the fetuses.

Fetal macrophages exhibit a M2 phenotype with enhanced remodeling and wound healing phenotype relative to adult mouse [41]. In addition, increased of M2 macrophages, as detected by expression markers (Arg1, Ccl17 and CD206), has been described during mouse lung development, which correlates with the stage of alveolar development, alveolar wall remodeling and microvascular maturation [42]. Of those M2 markers, Arg1 and CD206 are augmented by CS1 treatment indicative of a greater M2 infiltration in the damaged lung induced by this immunomodulator that may be also beneficial for lung development.

Recently, we described that atypical dual TLR2/4 ligands will skew macrophages to M2 differentiation, without being proinflammatory [29]. Moreover, TLR2 activation induces enzymes that synthesize vitamin A, mainly RALDH2, and promotes signaling by RAR in myeloid cells [25]. Likewise, RAR modifies the signal produced by TLR2 towards a monocyte capable of remodeling the extracellular matrix [43] and with M2 properties [44]. We observed an increase in several genes involved in the RA pathway upon treatment with the TLR2/4 ligand, CS1. This effect was evident both in macrophages in vitro and, notably, in the organs of nitrofen-affected embryos in vivo. Specifically, we found an upregulation of *Aldh1a2* that encodes RALDH2, a key enzyme in RA pathway which is expressed in developing lungs and catalyzes the synthesis of RA. Additionally, we observed an upregulation of RBP1 by CS1, the carrier protein involved in the transport of retinol in the cytoplasm before its conversion into RA by RALDH2. Both effects will lead to an increase in RA secretion by macrophages, similar to the induced in vitro by a TLR2 ligand [25]. Those CS1 activities were dependent of both TLR2 and TLR4, consistent with their described abilities to induce cytokine secretion of those types of atypical LPSs [29]. RA receptor genes (*Rara* and *Rarb*) have been linked to CDH [45, 46]. Knockout mice lacking these genes show significant lung hypoplasia and diaphragmatic defects [47]. Interestingly, these genes are upregulated in CS1 treated groups, suggesting CS1 treatment may contribute to the restoration of transcription of important genes in the RA pathways with a central role in the CDH recovery process.

Vitamin A plays an essential role in the embryonic development and its deficit may lead to CDH [46]. Besides, knockout of RBP (retinol binding protein) in mice significantly reduces retinol levels and causes embryonic abnormalities including pulmonary hypoplasia [48]. Retinol and RBP levels are also decreased in human newborns with CDH even though maternal RBP levels were comparable between mothers of CDH patients and mothers of healthy children [49], suggesting a specific defect in retinol homeostasis in the child rather than a maternal deficiency. Administration with RA has a protective effect in the nitrofen model of CDH [50]. Therefore, the observed upregulation of some RA pathway genes in CS1 treated groups suggests that these ligands have the potential to increase RA secretion in the lungs, potentially contributing to the therapeutic benefits of CS1 treatment in ameliorating CDH.

A recent study has shown that in mice TLRs are expressed in macrophages as early as E10.5 and that TLR ligands induce in these macrophages the ability to phagocytose apoptotic cells and secrete a broad spectrum of cytokines and chemokines [51]. Thus, embryonic macrophages can be receptive to TLR ligands and this may explain some of the effects we observed after treatment with CS1.

Interestingly, CS1 treatment is effective at least three days (at E12,5) after the induction of the teratogenic damage in rats or after the induction of the WT1 loss in the conditional WT1 mutant model. This finding is of great clinical significance because this embryonic stage (E12.5) in rats corresponds to over 20 weeks of gestation in human fetuses [52] and it is typically around this human pregnancy stage when the diaphragmatic hernia is first diagnosed in a routine echography [5, 6]. Therefore, the immunomodulatory treatment described here, could be used in human CDH cases, as effective treatment may be initiated at the time of diagnosis. Therefore, the favorable treatment window observed in the rat model provides promising prospects for clinical applications in human CDH treatment.

Side effects could be very important for a potential treatment during human pregnancy: This was not specifically assessed in this work and besides, the extreme pathological effects of nitrofen toxic treatment (rat model) or genetic deletion (mouse model), greatly difficult the analysis of the possible side effect of the treatment on the CDH fetuses. However, treatment with CS1 or CS2 was neither toxic to the normal pregnant mothers nor altered the healthy development of their pups. Importantly, in the mouse model, where siblings, either genetically altered on not (the efficacy of the genetic deletion is 70%) are present in in the same womb and received identical treatments, the 30% of genetically normal are born healthy, which support the low toxicity on the compounds on the fetuses. Future experiments, should evaluate if treated CDH- born animals are able to carry a normal life.

There is no etiological treatment for CDH, and in spite of pharmacological and surgical measures, aiming to reduce the pulmonary hypertension and to close the diaphragm repositioning the affected organs into the abdomen, the overall survival remains alarmingly low [6]. Unfortunately, the complex and multifactorial nature of the disease, with no single genetic cause, makes genetic therapies basically impractical. In this context, targeting the immune system to promote tissue repair in CDH represents an innovative and promising avenue of research.

A potential limitation of our study is that human CDH is a complex multifactorial disease, and no all factors have been taken into account in our studies. So, we were unsure if immunomodulator treatment will work for all types of CDH. Another related limitation is how the stage and extent of hernia damage will affect the treatment. Trying to partially avoid those limitations, we studied the 2 models: nitrofen in rats and WT1 deletion in mice, which produce very severe hernias. Nonetheless, the potential application of these immunomodulators in human CDH treatment, particularly during the early stages of gestation, holds great promise for improving outcomes and addressing the pressing medical need in this challenging condition. In support of this, the TLR2/4 dual ligand CS1 has been recently awarded the orphan drug designation by the European Medicines Agency (EMA) for the treatment of CDH (https://ec.europa.eu/health/documents/community-register/html/o2801.htm.)

## Conclusion

We believe that the most significant result of this study is the fact that a single dose of new immunomodulators, repairs developmental problems in CDH, including those derived from a genetic cause. Our results, demonstrate the effectiveness of immunomodulators that influence macrophage activation in rescuing CDH in both teratogen-induced and genetic mouse models, being a promising solution for CDH. The TLR2/4 ligand CS1 induces the infiltration of M2 macrophages, and the expression of retinoic acid pathway genes in damaged organs that in turn would be responsible for repairing the damage allowing the closure of the hernia in the diaphragm. They also enhance proper lung development being nontoxic for their mothers or their pups. Overall, our study highlights the promising therapeutic potential of immune modulator CS1 for the treatment of CDH.

## Supplementary information

**Additional file 1**.

## Data Availability

Not applicable.

## Abbreviations

CDH: Congenital diaphragmatic hernia
LPS: Lipopolysaccharide
PHMP: Posthepatic mesenchymal plate
PPFs: Pleuroperitoneal folds
RA: Retinoic acid
ST: Septum transversum
TLR: Toll like receptor
