# Supplementary material for: Toll-like receptors ligand immunomodulators for the treatment congenital diaphragmatic hernia
Source: Orphanet J Rare Dis. 2024 Oct 18;19:386. doi: 10.1186/s13023-024-03384-7 (PMC11487987; doi:10.1186/s13023-024-03384-7)
Supplement: Supplementary file 1 — Additional file 1. [file 13023_2024_3384_MOESM1_ESM.pdf]

Table S1 Rat RT-PCR primers

Rat primers	Forward	Reverse
Rara	TTGGAATGGCTCAAACCAC	AGGGCTGGGCACTATCTCTT
Rarb	ATCGATGCCACCTCTCATTC	GGGGTCAAGGGTTCATGTC
Pparg	CCCAATGGTTGCTGATTACA	GGACGCAGGCTCTACTTTGA
Ccl2	AGCATCCACGTGCTGTCTC	GATCATCTTGCCAGTGAATGAG
Stra6	CTCTGGTCTTGTCCTGCTCA	TGCTTTAAGATTGGCCCTGT
Zfpm2 (Fog2*)*	GTATTTTCTTTCGCCGATGC	TTGCATTTAACAGCCAAGGTT
Nr2f2	CCTCAAGGCCATAGTCCTGT	CTTTCCACGTGGGCTACATC
Aldh1a2	TCTCATGGTGTCCTCTGCAA	TCTGAGCATTTAAGGCGTTG
Wt1	GAACCTCGGAGCTACCTTGA	TCTGCCCTTCTGTCCATTTC
Rbp1	AGGCATAGATGACCGCAAGT	CACTGAAGCTTGTCACCATCC
Slit3	GCTGGTGTATGACAGCCTGA	CCAGGTTCAGGGTCTGGTT
Gadph	TCAAGAAGGTGGTGAAGCAG	AGGTGGAAGAATGGGAGTTG
Ifng	TTTTGCAGCTCTGCCTCAT	AGCATCCATGCTACTTGAGTTAAA
Il12a	CAGGCCATAAATGCAGCAC	CCGCTGTGATTCAGAGACC
ll10	AGTGGAGCAGGTGAAGAATGA	TCATGGCCTTGTAGACACCTT
Arg1	CCGCAGCATTAAGGAAAGC	CCCGTGGTCTCTCACATTG
Foxp3	GTACAGCCGGACACACTGC	GCTGACTTCCAAGTCTCGTGT
Il17a	CTTCACCCTGGACTCTGAGC	CCTCAGCGTTGACACAGC
mRPL13A	TCCCTCCACCCTATGACAA	GCCCCAGGTAAGCAAACTT

Table S2 Mouse RT-PCR primers

Mouse primers	Forward	Reverse
Arg1	AGAGATTATCGGAGCGCCTT	TTTTTCCAGCAGACCAGCTT
Aldh1a2	ATCGCTTCTCACATCGGCAT	ACAGCGTAGTCCAAGTCAGC
Rbp1	ATAGACGACCGCAAGTGCAT	CTCAGCTCTCATTTCCAGGTGA
Ccl2	AGTCTCTGCCGCCCTTCT	GTGACTGGGGCATTGATTG
Il12	TGGTTGCCATCGTTTTCCTG	ACAGGTGAGGTTCACTGTTTCT
Slit3	GCCACAAGGATGGGTTCA	AGTCATCTGGGTTGATCTCACA

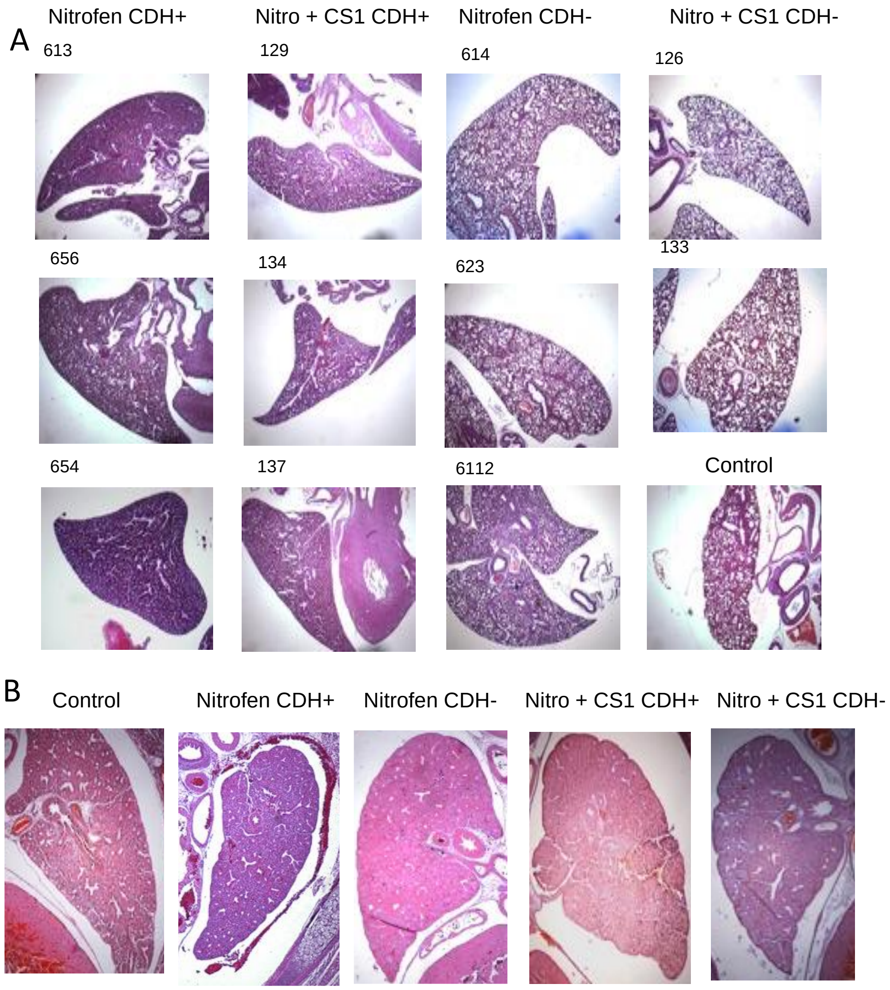

Figure S1. Effect of TLR ligand in the alterations in lung development induced by nitrofen. Pregnant Wistar rat females were treated with nitrofen at E9.5 and 3 days later injected intraperitoneally with 100 µg/Kg CS1 . A) Hematoxylin-eosin images of E21 lungs of 3 different fetuses from the different groups. B) A representative image from each group at E18.

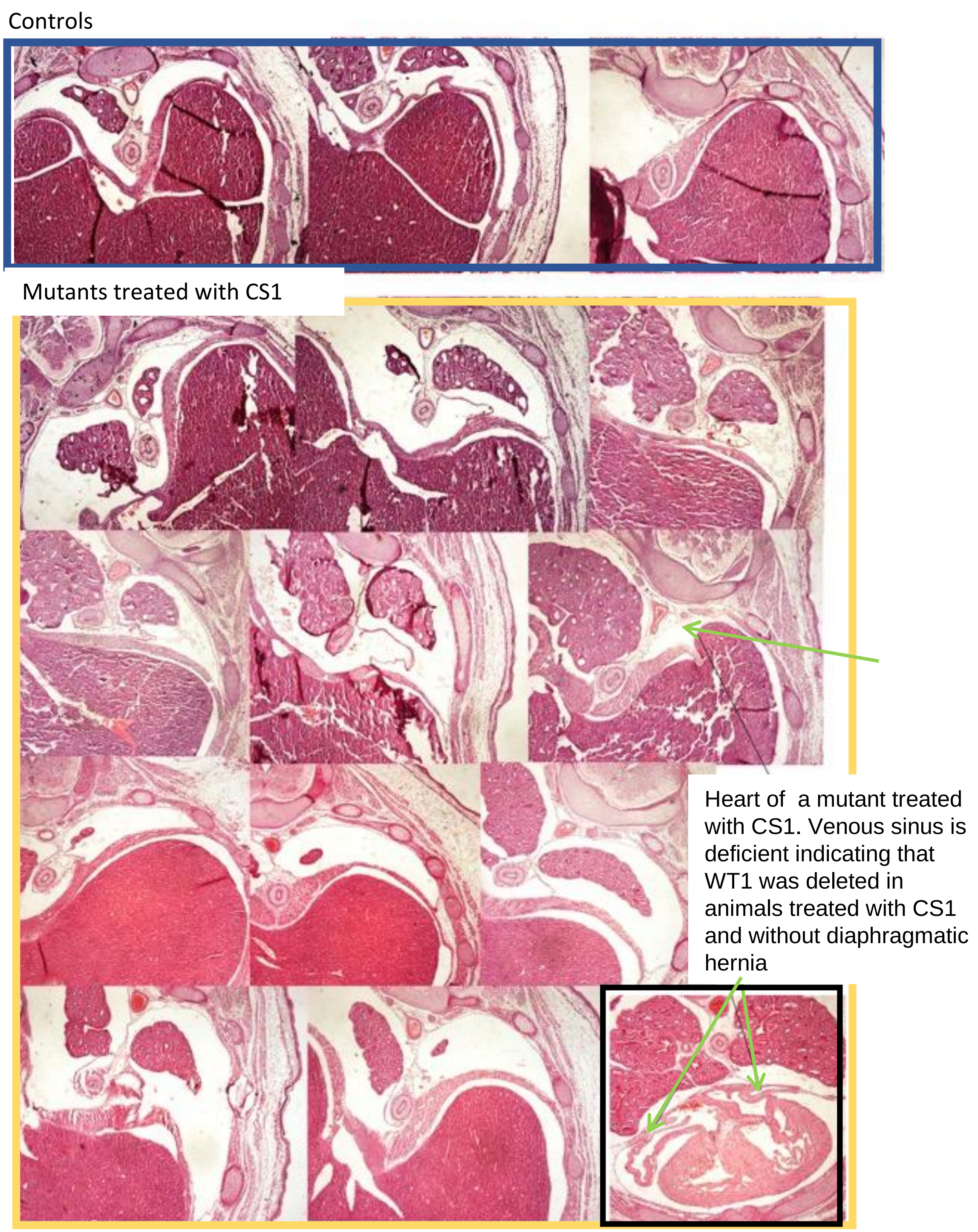

Figure S2 Effect of CS1 TLR ligand in diaphragmatic hernia of G2-GATA4^{Cre};Wt1$^{fl/fl}$ mice . Pregnant mice mothers were treated with CS1 or PBS intraperitoneally twice at E9.5 and E10.5. Embryos were analysed at E15.5 . Images of the diaphragm from the 11 WT1 mutant embryos of mothers treated CS1 obtained. The heart images of a Nitro+CS1 mutant CDH- is also shown. Embryos (3) from control mothers are also shown.

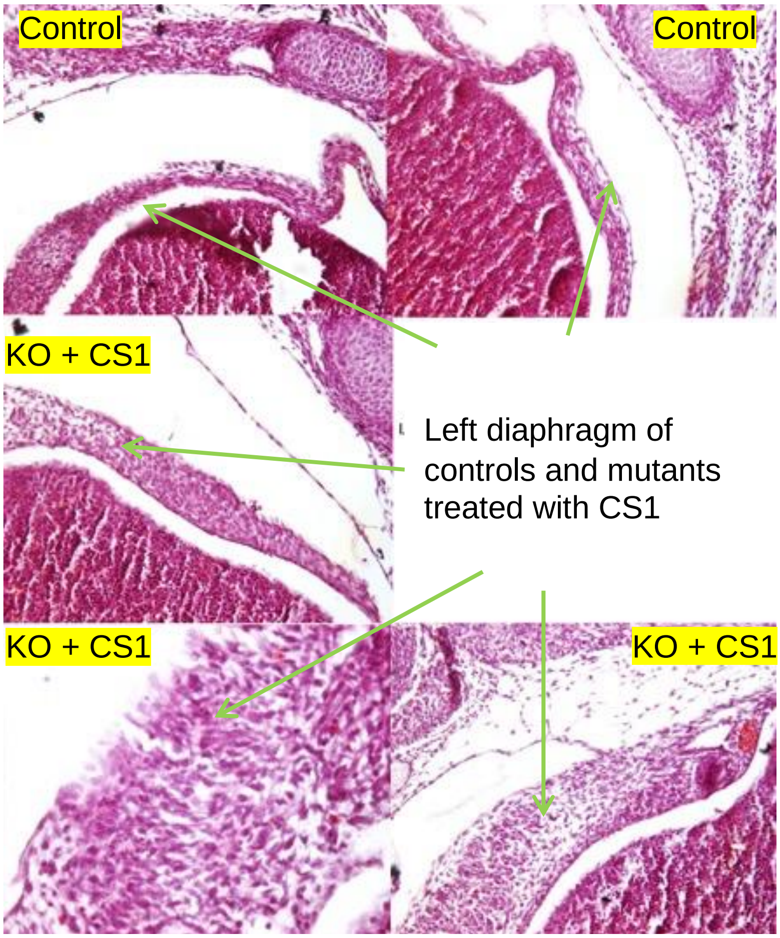

Figure S3 Effect of CS1 TLR ligan in diaphragmatic hernia of G2-GATA4^{Cre};Wt1$^{fl/fl}$ mice. Pregnant mice mothers were treated with CS1 or PBS intraperitoneally twice at E9.5 and E10.5. Embryos were analysed at E15.5. Representative images of the diaphragm from WT1 mutant embryos of mothers treated CS1 (3) and from control CS1 treated (2) mothers are shown
